# Supplementary material for: Molecular Mechanism of Mutational Disruption of DCLK1 Autoinhibition Provides a Rationale for Inhibitor Screening
Source: Int J Mol Sci. 2023 Sep 13;24(18):14020. doi: 10.3390/ijms241814020 (PMC10531055; doi:10.3390/ijms241814020)
Supplement: Supplementary file 1 [file ijms-24-14020-s001.zip › ijms-2508175-supplementary.pdf]

Supplementary Material

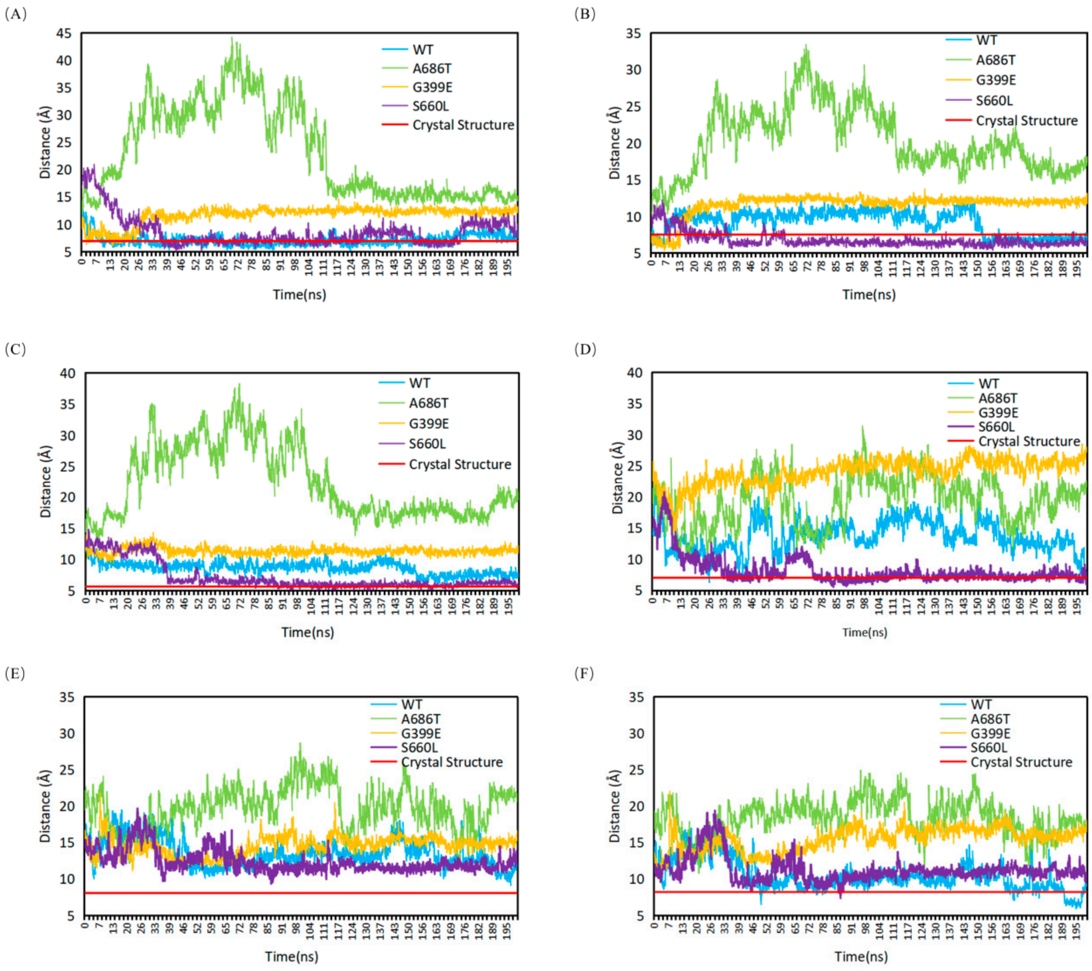

Supplemental Figure S1 Variations of selected distances between the Cα atoms of the amino acids with simulation time. The red horizontal line is the distance value in the crystal structure, and the blue, green, orange and purple curves represent the wild type, A686T mutant, G399E mutant and S660L mutant respectively. (A) to （E） show the change of the distance of the Cα atoms between V682-L467, V684-L518, I685-M465, V690-I433, K692-D533, K692-D511 during the simulations. Because of the change of hydrophobicity, the variation of A686T mutant is generally larger, and the fluctuation of residues in R3 region(V690-I433) is higher than that in R2 region. It is speculated that the multi-turn area on the N end of R2 helix plays a role in the stability of the structure of the R2 and in its binding with KD.

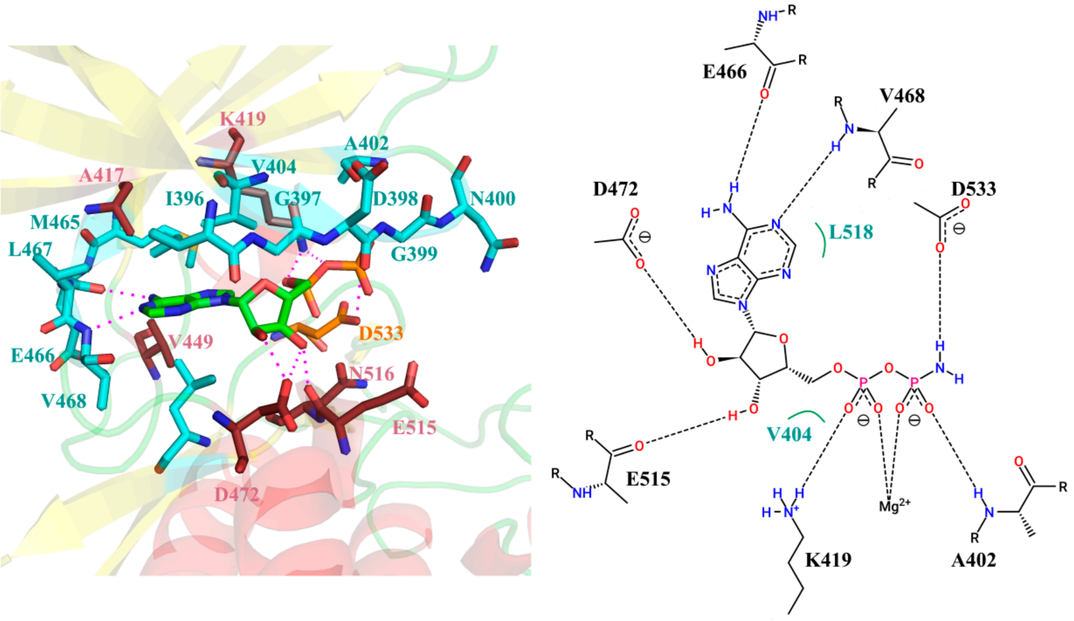

Supplemental Figure S2 The structure and the interaction pattern of DCLK1 with AMPPN(PDB ID: 5JZJ). The amino group of adenine forms a hydrogen bond with the carbonyl of the backbone of E466, N_1 forms a hydrogen bond with the amide nitrogen of the backbone of V468, and the imidazole ring forms a hydrogen bond with the methyl group of V404. The oxygen and hydroxyl groups of the ribose ring form a hydrogen bond network with D472, G397, and E515, and the phosphate group forms hydrogen bonds with K419, G399, A402, and D533.

PDB ID	Kinase	Ligand HET-code	IFP similarity
6ZLN	CLK1	PKB	0.83
4XG9	SYK	X9G*	0.82
6YID	ULK1	EDJ*	0.80
2C5Y	CDK2	MTW	0.79
2R3F	CDK2	SC8	0.79
2R3Q	CDK2	5SC	0.79
2VV9	CDK2	IM9*	0.79
5T1H	CSNK2A1	75E	0.77
1OIQ	CDK2	HDU	0.76
4HGS	CSNK1G3	15G	0.76
6PJX	GRK5	SGV*	0.76
6NZH	TYK2-b	L9A	0.75
5L2S	CDK6	6ZV*	0.75
7AW0	MERTK	S4Q	0.75

Supplemental Table S1 The small molecules obtained from preliminary screening in the KLIFS database. Small molecules marked with * can form hydrogen bonds with D533 after docking.

PDB ID	Kinase	Ligand HET-code	IFP similarity	Structure of the ligand
4XG9	SYK	X9G	0.82	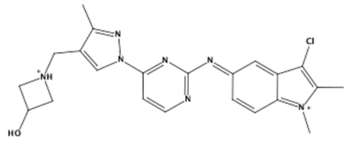
2VV9	CDK2	IM9	0.79	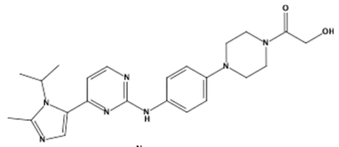
6PJX	GRK5	SGV	0.76	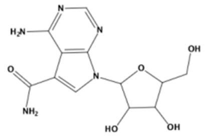
5L2S	CDK6	6ZV	0.75	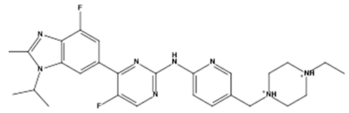

Supplemental Table S2 The four more plausible small molecules screened by the binding mode with DCLK1 after the docking, especially whether it can occupy the binding site of D533.
